# Novel Copper Oxide Bio-Nanocrystals to Target Outer Membrane Lectin of Vancomycin-Resistant *Enterococcus faecium* (VREfm): In Silico, Bioavailability, Antimicrobial, and Anticancer Potential

**DOI:** 10.3390/molecules27227957

**Published:** 2022-11-17

**Authors:** Mahmoud Kandeel, Mohamed Sharaf, Arshad Mahdi Hamad, Ahmad O. Babalghith, Mohnad Abdalla, Muhammad Arif, Reem Binsuwaidan, Nashwah G. M. Attallah, Hossam Aladl Aladl Aladl, Samy Selim, Mariusz Jaremko

**Affiliations:** 1 Department of Biomedical Sciences, College of Veterinary Medicine, King Faisal University, Al-Hofuf 31982, Saudi Arabia; 2 Department of Pharmacology, Faculty of Veterinary Medicine, Kafrelshikh University, Kafrelshikh 33516, Egypt; 3 Department of Biochemistry, Faculty of Agriculture, AL-Azhar University, Nasr City, Cairo 11651, Egypt; 4 Department of Biochemistry and Molecular Biology, College of Marine Life Sciences, Ocean University of China, Qingdao 266003, China; 5 Department of Biology, College of Science, Tikrit University, Tikrit 34001, Iraq; 6 Medical Genetics Department, College of Medicine, Umm Al-Qura University, Mecca 24382, Saudi Arabia; 7 Pediatric Research Institute, Children’s Hospital Affiliated to Shandong University, Jinan 250022, China; 8 Department of Pharmaceutical Sciences, College of Pharmacy, Princess Nourah Bint Abdulrahman University, Riyadh 11671, Saudi Arabia; 9 Egyptian Drug Authority (EDA) Previously NODCAR, Giza 12513, Egypt; 10 Department of Internal Medicine, Faculty of Medicine, AL-Azhar University, Nasr City, Cairo 11651, Egypt; 11 Department of Clinical Laboratory Sciences, College of Applied Medical Sciences, Jouf University, Sakaka 72388, Saudi Arabia; 12 Smart-Health Initiative and Red Sea Research Center, Division of Biological and Environmental Sciences and Engineering, King Abdullah University of Science and Technology, Thuwal 23955-6900, Saudi Arabia

**Keywords:** nanocrystal, *Olea europaea*, *Enterococcus faecium*, green biosynthesis, antimicrobial, anti-cancer, molecular docking, molecular dynamic simulation

## Abstract

In present study, we used *Olea europaea* leaf extract to biosynthesize in situ Copper Oxide nanocrystals (*CuO @OVLe NCs*) with powerful antibacterial and anti-cancer capabilities. Physio-chemical analyses, such as UV/Vis, FTIR, XRD, EDX, SEM, and TEM, were applied to characterize *CuO @OVLe NCs*. The UV/Vis spectrum demonstrated a strong peak at 345 nm. Furthermore, FTIR, XRD, and EDX validated the coating operation’s contact with colloidal *CuO @OVLe NCs*. According to TEM and SEM analyses, *CuO @OVLe NCs* exhibited a spherical shape and uniform distribution of size with aggregation, for an average size of ~75 nm. The nanoparticles demonstrated a considerable antibacterial effect against *E. faecium* bacterial growth, as well as an increased inhibition rate in a dose-dependent manner on the MCF-7, PC3, and HpeG2 cancer cell lines and a decreased inhibition rate on WRL-68. Molecular docking and MD simulation were used to demonstrate the high binding affinity of a ligand (Oleuropein) toward the lectin receptor complex of the outer membrane to vancomycin-resistant *E. faecium* (VREfm) via amino acids (Leu 195, Thr 288, His 165, and Ser 196). Hence, our results expand the accessibility of *OVLe’s* bioactive components as a promising natural source for the manufacture of physiologically active components and the creation of green biosynthesis of metal nanocrystals.

## 1. Introduction

Antibiotics have saved many lives over the last 100 years, and experts once believed that humans might eradicate illnesses caused by bacteria. On the other hand, antibiotic misuse has steadily built resistance, resulting in the eradication of antibiotics after decades of usage and the emergence of several multidrug-resistant microorganism strains that are insensitive to conventional antibacterial strategies, such as *E. faecium* [1]. Drug resistance is a significant danger linked with microbial infections, leading to treatment failure. Currently, various health organizations throughout the globe are warning that multidrug-resistant microorganisms are a serious hazard since they risk thousands of lives and have an unquantifiable cost [2,3,4,5,6].

Vancomycin-resistant *Enterococcus faecium* (VREfm) is a gram-positive aggressive pathogen that often infects the human and animal oral cavity, digestive tract, and genital tract. VREfm has no negative implications for the host in a healthy individual. Unfortunately, in immunocompromised people, the pathogen can induce life-threatening diseases [7,8].

Antibiotic overuse in recent years has accelerated the growth of antibiotic-resistant bacterial strains (particularly vancomycin-resistant strains), resulting in their predominance in human and animal germs [9]. In recent years, managing infections induced by VREfm has become extremely challenging due to the frequency of multidrug-resistant *E. faecium* strains, particularly vancomycin-resistant strains, generating severe concerns among healthcare professionals [10]. A thick layer of peptidoglycan typically surrounds gram-positive microorganisms, which suggests that in *E. faecalis*, peptidoglycan alteration by secondary cell wall polymers is crucial for evading complement identification [11]. *E. faecalis* secondary cell wall polymers are required for tolerance to complement stimulation by coupling with lectin [12].

As a result, new treatment alternatives that are less successful at selecting for resistance, such as plant-derived phenolic chemicals, are necessary. Nano-antibiotics have broad antibacterial efficacy against bacteria, protozoa, and fungi. Metallic nanoparticles have received significant attention since metals have inherent antibacterial properties and display antimicrobial effect regardless of microbe drug sensitivity profiles. Furthermore, nanoparticles may increase the efficacy of currently available antimicrobial drugs [13].

Plants have medical significance because they include chemical substances that perform a particular physiological function in the human body. Phenolic compounds, alkaloids, tannins, and flavonoids are the major significant bioactive plant substrates [14]. Because of their polyphenol antioxidant and anti-inflammatory properties, certain phytochemicals can greatly lower antibacterial and cancer risk. Preclinical research suggests that phytochemicals can help prevent breast, colorectal, and other cancers [15].

Metal nanoparticles are synthesised using plant materials such as leaves, roots, latex, seeds, and stems [16,17]. Techniques for producing nanoparticles using naturally available reagents such as plant extracts may be appealing for nanotechnology. Furthermore, they can be utilised to minimise the toxicity of a medicinal medicine [18].

**Figure 1:**
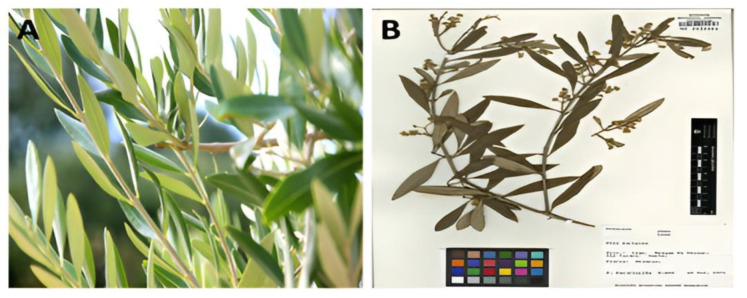
(**A**) *Olea europaea L. folium* (Oleaceae) leaves; (**B**) Botanical Voucher Specimen, MOBOT, Tropicos.org (http://www.tropicos.org/Image/100000992, accessed on: 1 April 2022).

The olive tree’s (*Olea europaea*, Oleaceae) leaves (Figure 1A,B) are widely employed in traditional herbal therapies to avoid and treat a wide range of diseases, including antimicrobial, diabetes, cardiovascular disease, cancer, and several health problems, because they contain several critical antioxidant and phenolic components to avoid oxidative damage [19,20,21]. Oleuropein, tyrosol, and hydroxytyrosol, in addition to caffeic acid and ligstroside, are antioxidant compounds found in the olive tree that have been linked to the prevention of several illnesses [22]. Oleuropein, a naturally occurring compound of the secoiridoid group, is the primary active ingredient in olive leaf and its extract. Several investigations have shown that oleuropein possesses a range of pharmaceutical and health-promoting properties, including antispasmodic, immune-stimulating, cardioprotective, hypotensive, anti-inflammatory, antioxidant, anti-cancer, and antithrombotic properties [20,23].

In present study, we modified the method of preparation of Copper Oxide NPs using olive leaf extract at room temperature (25–27 °C) in order to preserve the active compounds of the plant extract and to avoid breaking them at high temperatures during preparation as in other methods mentioned previously (100 °C) [24], which increases their effectiveness. As a result, the purpose of this research was to examine the size-controlled compounds to biosynthesize in situ nanoscale Copper Bio-nanocrystals Oxide (CuO NCs) used *Olea europaea* leaf extract and surface functionalization using in situ oxidation–precipitation techniques, together with the exploration of the antimicrobial, and anticancer efficiency. Moreover, this is the first publication to identify the phytochemicals substrate (oleuropein) antimicrobial properties of vancomycin-resistant *Enterococcus faecium* (*VREfm*) via greatly improving their interactions with lectin outer membrane protein.

## 2. Results and Discussion

### 2.1. Role of Phyto-Reductants and Capping of OVLe Agents for Tailoring and Characterization of OVLe@CuO NCs

According to earlier investigations, the oleuropein exists in high concentrations compared to other phenolic components as observed by the HPLC chromatogram of olive leaf extract [25,26]. Oleuropein, a highly unstable molecule that quickly breaks down into the more polarizable hydroxytyrosol (C_8_H_10_O_3_) in organic solvents, may have played a role in the capping action CuO-NS underwent in the current work to modify high-energy planes (atoms). During the synthesis of NS, it may concurrently function as a phyto-reducing and phyto-capping agent [27].

#### 2.1.1. UV-Vis Absorption Spectroscopy Analysis

**Figure 2:**
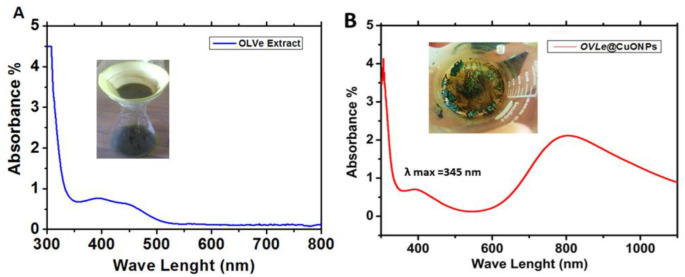
UV–Vis absorption spectra: (**A**) Olive leaf extract (OVLe), (**B**) *CuO @OVLe NCs*.

As the olive leaf extract, the absorption peak gets more sharpness and blue shift was observed from 400 to 458 nm (Figure 2A). The copper salt, which is reduced by the ions to create nanoparticles, forms complexes with the phytochemicals in the extracts. Therefore, we employed UV-Vis spectroscopy in the 200–1100 nm range to examine the color change in the produced Solutions. Due to the *CuO @OVLe NCs* ‘ core electrons’ inter-band transition, Figure 2B shows a notable peak at 345 nm. These findings resembled Aziz et al. [28]. For the synthesis of *CuO @OVLe NCs*, Mahmoud et al. 2021 also employed orange peel and mint leaf extract, and they discovered their absorption peak at 325 nm [29].

#### 2.1.2. FTIR Analysis

**Figure 3:**
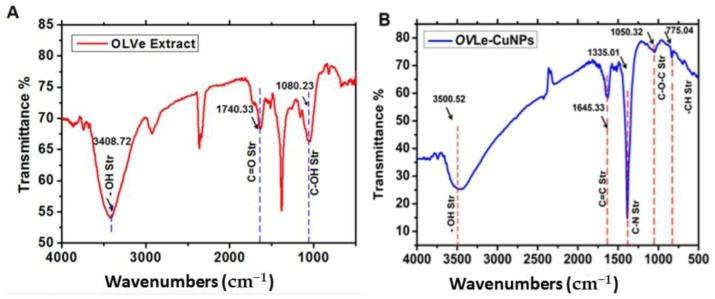
FTIR spectra: (**A**) Olive leaf extract (OVLe), (**B**) *CuO @OVLe NCs*.

As a consequence, we noticed that the FTIR vibration varied depending on the functional groups. These groups have a distinctive absorption in the IR region that causes the compound’s energy bonds to stretch and curve more in the peaks. The FTIR spectra revealed the presence of different functional groups. The IR bands (Figure 3A) observed at 3408.72 and 1740.33 cm^−1^ in dried olive leaf were characteristic of the O-H and C=O stretching modes for the OH and C=O groups, possibly of oleuropein, apigenin-7-glucoside, and/or luteolin-7-glucoside. The very strong band at 1080.23 cm^−1^ could be assigned to the C-OH vibrations of the protein in the olive leaf. These results are close to those mentioned by Khalil et al. [30]. In addition, the surface chemical coordinates and chemical composition of the *CuO @OVLe NCs* were examined by FTIR. The functional groups in the *CuO @OVLe NCs* were identified using the peaks shown in Figure 3B. The C-O, O-H, and H-H bond order frequencies are seen in the vibration modes between 2500 and 4000 cm^−1^ [27]. The identified Cu-O peak locations were at 592, 607, and 665 cm^−1^, whereas 453 cm^−1^ was discovered for the Cu-O stretching bond frequency. The findings related to Cu-O are consistent with earlier studies [31,32].

#### 2.1.3. XRD Analysis

**Figure 4:**
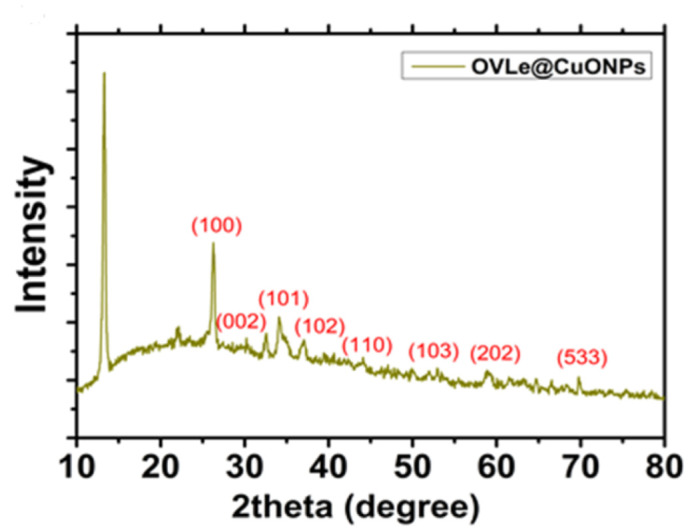
XRD of *CuO @OVLe NCs*.

XRD patterns were used to determine the materials’ structure and phase purity, as illustrated in Figure 4. Overall, 2θ values of 67.79° (533), 64.84° (202), 55.23° (201), 54.50° (112), 52.50° (200), 51.52° (103), 46.33° (110), 39.29° (102), 36.33° (101), 29.01° (002), and 26.3° (100) degrees, the strong diffraction peaks were seen. When compared to the information from stander values (JCPDS card No. 48-1548), the diffraction peaks of XRD are extremely well matched with those found by Siddiqui et al. [33]. The produced *CuO @OVLe NCs* were confirmed by each reflection peak. This pattern adheres to the International Centre for Diffraction Data’s normative peaks. In addition, the wide breadth of the diffraction bands in X-ray diffraction patterns indicated tiny particle sizes. These results were expected based on previous studies by Padil et al. [34].

#### 2.1.4. Surface Morphology

**Figure 5:**
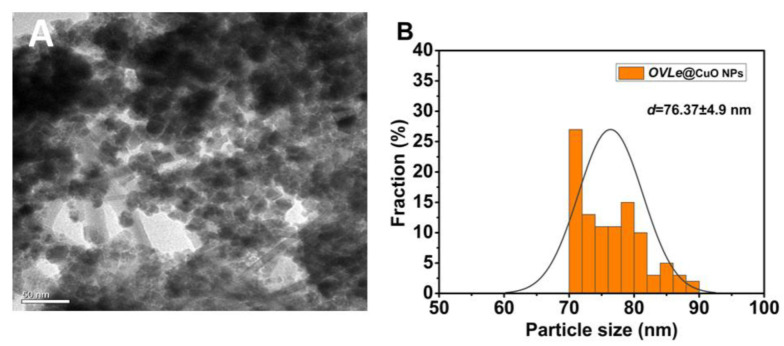
(**A**) Average particle size of prepared *CuO @OVLe NCs* evaluated utilising TEM (scale bar = 50 nm). (**B**) The size distribution of prepared *CuO @OVLe NCs* data is shown as the mean ± SD (*n* = 3).

**Figure 6:**
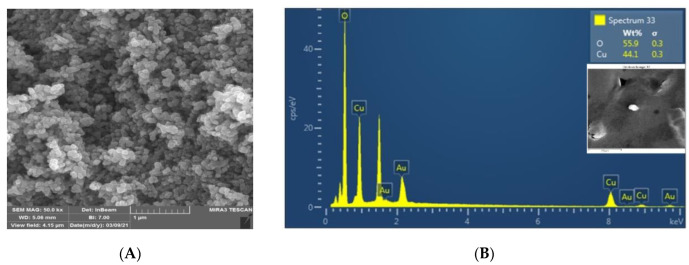
(**A**) The shape of synthesized *CuO @OVLe NCs* assessed utilising SEM (scale bar = 1 µm); (**B**) EDX profile.

SEM analysis was used to determine the *CuO @OVLe NCs’* size, shape, and surface morphology. Figure 5A,B and Figure 6A, respectively, exhibit TEM and SEM images. According to certain structural properties, the synthesised products had spherical and crystalline structures and diameters of 76.37 ± 4.9 nm. Overall, previous findings support the notion that producing CuO NCs using an aqueous leaf extract of *OVLe* is an efficient and environmentally benign method. The findings support numerous previous investigations, as shown in our current investigation [32]. Energy dispersive X-ray spectroscopy (EDX) was used to determine the elements that comprise the biogenic *CuO @OVLe NCs*. Figure 6B displays the results of an EDX investigation that identified copper (44.08%) and oxygen (55.92%). A high peak at 0.93 keV and a peak at 8.24 keV were also seen, both of which indicated the presence of copper (Cu), while a peak at 0.473 keV was linked to the presence of oxygen (O). Previous studies have shown that a number of EDX-discovered elements, including Si, Au, and Cl, act as capping agents for biogenic CuO NPs. The reactivity, surface area, rate of absorption into microbial cells, cell surface contact, protein binding, and other properties of NPs are determined by their size and shape [35], and these parameters are of prime importance for antimicrobial applications. These results indicate that the designed nanoparticles of formulations around ~75 nm were small enough to satisfy the steric prerequisite for rapid diffusion through the bacteria’s outer membrane. These results confirmed the successful biosynthesis of copper nanoparticles. It was also found that the bio coating shell process for CuO NPs did not affect the phase change of copper oxide.

### 2.2. Antibacterial Potential of CuO @OVLe NCs

**Figure 7:**
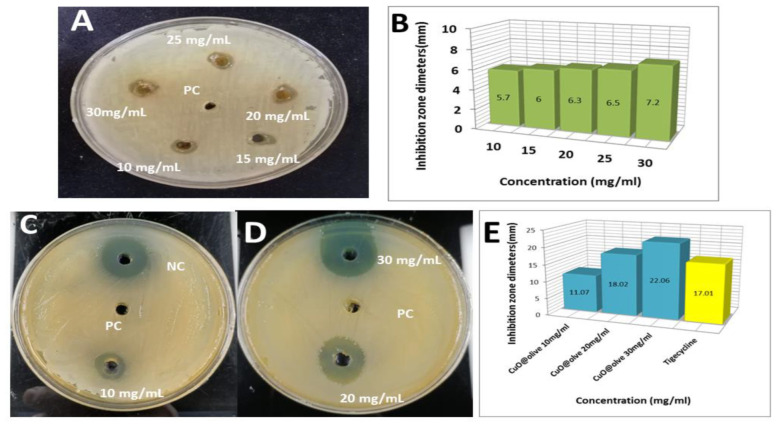
(**A**) Zone of inhibition (mm) plate with diverse concentrations 10, 15, 20, 25, and 30 mg/mL of OVLe; (**B**) *Enterococcus faecium* bacterial growth curve; (**C**,**D**) zone of inhibition (mm) plate with diverse concentrations 10, 20, and 30 mg/mL of *CuO @OVLe NCs*; (**E**) *Enterococcus faecium* bacterial growth curve.

*OVLe* and *CuO @OVLe NCs* suspensions at different concentrations were analysed for antibacterial activity against *VREfm* cultured on Muller-Hinton agar medium at 37 °C for 24 h using a disc diffusion assay. The ability of the antibacterial agent (NPs) to rupture the bacterial cells is shown in Table 1 and Figure 7.

*OVLe* showed mild inhibitory action, with a maximum zone of the inhibition region mainly depending on the concentration of 7.2 mm against *VREfm* for high concentrations of 30 mg/mL/disk (Figure 7A,B). No zone of inhibition was detected for the positive control (DIH_2_O) on the disk of *VREfm*. Tigecycline was determined as a negative control which recorded an inhibition zone diameter of 17.01 mm (Figure 7C–E), These results are in agreement with that of Latifa et al. [36], who found that the antibacterial efficiency of isolates produced resistance results of *VREfm* (zone diameter ≤ 15 mm) utilising the disk diffusion assay.

The quantitative evaluation of antibacterial *CuO @OVLe NCs* by diffusion in the pits was performed, and it was observed that the inhibition region mainly depended on the concentration. However, the *VREfm* strain exhibited the highest sensitivity to *CuO @OVLe NCs* at the concentrations of 30 mg/mL/disk and recorded inhibition zone diameters of 22.06 mm (Figure 7C,E), which may have been due to the divergent physio-chemical characteristics of *CuO @OVLe NCs*. In addition, *CuO @OVLe NCs* showed mild inhibitory action with a maximum zone of inhibition of 11.07 mm and 18.02 mm for *CuO @OVLe NCs* against *VREfm* for differential concentrations of 10 and 20 mg/mL/disk, respectively (Figure 7C–E). The multimode actions that green fabricated *CuO @OVLe NCs* caused the complete growth inhibition of all *VREfm* bacterial strains.

Cu has shown varied inhibitory activity due to its various physio-chemical properties. Olive leaf extract phyto-reductants have also demonstrated modest inhibitory activities, and the bactericidal capabilities of olive plant extract are extensively documented in the literature [26,37]. The observed differential in the zone of inhibition across bacterial strains might be attributed to gram-negative pathogens having structural changes in their cell walls, such as the deposition of lipopolysaccharide material [38], the velocity of NS diffusion through the chemical properties, bacterial envelop, ionic discharge, binding activation energy, and surface charge attraction, which increases their pathogenicity [39]. Cu has a changeable oxidation state that alternates between Cu^+^ and Cu^2+^, which can be attributed to the fact that most transition metals can include either 3d or 4s shells for electron shuffling [40]. When metal ions with varying oxidation states are exposed to the biological environment, they can cause deadly ROS. Even more reactive and heavily contributing to the production of ROS are metal ions from NPs. According to the theory in Figure 6, the antibacterial action in our investigation may also be closely related to the photoactivation of ROS by NPs on bacterial growth [41]. Furthermore, the cellular environment is affected by lethal ROS species (OH^−^, _1_O^2−^, and ^*^O_2_), which increases oxidative stress. Direct genome destruction, ROS-induced mitochondrial damage, plasmid and cell protein denaturation, and partial or complete loss of cell wall permeability are mainly induced by the upregulation in oxidative stress [42].

### 2.3. Screening Cytotoxicity Study and Anti-Cancer of CuO @OVLe NCs

**Figure 8:**
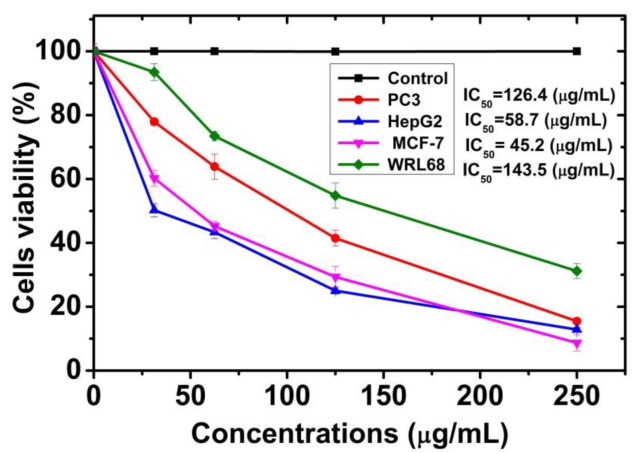
Viability of normal WRL68 cell and cancer cells (PC3, HepG2, and MCF-7), cells treated with different concentrations of *CuO @OVLe NCs*. Numerical data are described as the mean ± SD (*n* = 3).

Both the normal cell line (WRL-68) and the cancer cell line (MCF-7, PC3, and HpeG2) were treated with concentrations ranging between 31.25 and 250 µg/mL of *CuO @OVLe NCs* (Figure 8) for 24 h at a temperature of 37 °C at a rate of three replicates for each concentration. The control sample without treatment was used for comparison. The extent of the toxicological effect was also evaluated by extracting the percentage of the growth inhibition rate compared to the control (100% growth). The 3T3 Phototox software was used to estimate the IC_50_ of prepared samples in different cell lines using the absorbance values disclosed in the following capture of the red dye and the appropriate amounts of the *CuO @OVLe NCs* employed in the cytotoxicity studies. The highest quantities of *CuO @OVLe NCs* examined in this study showed minimal toxicity to the normal cell line WRL-68 and to cancer cells (PC3, HpeG2, and MCF-7) (Figure 8). They increased the inhibition rate in a dose-dependent manner of MCF-7 cells (IC_50_, approximately 45.24 µg/mL), PC3 cells (IC_50_, approximately 126.40 µg/mL), and HpeG2 cells (IC_50_, approximately 58.74 µg/mL), and increased the cell viability and decreased the inhibition rate of WRL-68 at a high dose (IC_50_, approximately 143.50 µg/mL). Biresaw and Taneja (2022) found approximately similar results when studying Copper nanoparticles with green synthesis derived from *Prunus nepalensis* phytochemicals [43]. Furthermore, CuNPs synthesized from different plant extracts, such as *Ficus religiosa* [44], indicated cytotoxic activities on breast (MCF-7) cancer cell lines and human liver carcinoma cells (HepG2) in a concentration-dependent manner, which was probably mediated through ROS generation and oxidative stress. Our findings may be due to the level of glycolipids and glycopeptides increasing significantly in the cell wall of cancer cells compared to normal cells, as these substances act as special receptors. The resulting Cu ion can interact large biomolecules such as enzymes, DNA, and RNA, inducing the oxidation process, and cause damage to the mitochondria [45]. In addition, green CuNPs nanoparticles affect the cancer cell, inhibiting the respiratory chain and stopping ATP synthesis [46]. Moreover, the effect on the cancerous line compared to the natural one, such as the MCF-7 cancer line and HepG2, was due to the selectivity to some metabolic properties that cancer cells possess and their absence in normal cells, such as their metabolic nature, the receptor shape on the surface of the cancer cells, and the affinity to bind to various compounds [47,48,49]. Further, it has been reported that green synthesized CuO NPs exhibit a low cytotoxic nature and genotoxic toward normal somatic cells compared to NPs produced by other chemical or physical approaches [50,51]. Doped or undoped CuO NPs engineered by different chemical techniques also demonstrated cytotoxicity to healthy cells [52].

### 2.4. Molecular Docking and Interaction of Ligands with the Active Site of Enterococcus faecium Lectin

**Figure 9:**
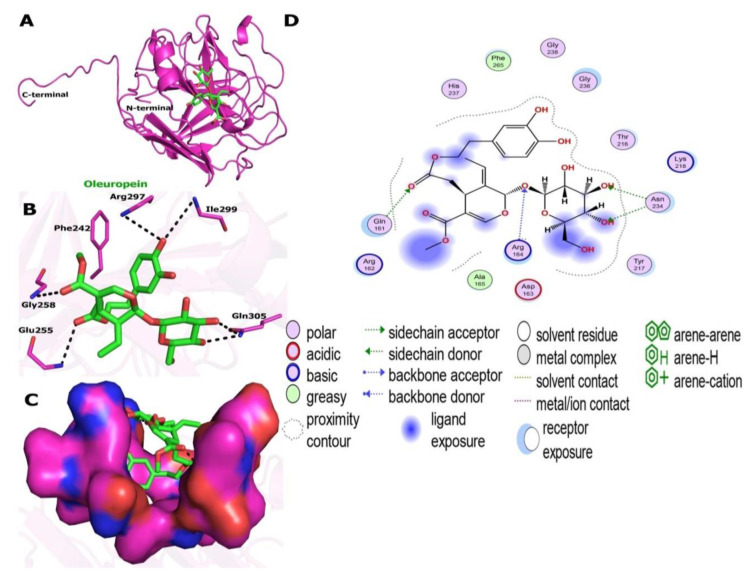
Molecular docking study of oleuropein toward the binding pocket of *EFLec*. (**A**) The surface representation of the active-site flap of *EFLec* with ligand, shown at the entrance of the binding pocket. (**B**) Amino acids involved in a hydrogen bond with oleuropein are shown as sticks. PyMol created these figures. Oleuropein (cyans), the crystal structure of *EFLec* (NCBI ID WP_033741131.1) (greens), and oxygen atoms are presented in red, and nitrogen atoms are shown in blue and purple dashed lines assigned to the bonding interactions. (**C**) Electrostatic surface of *EFLec*. Positive charges are in blue, negative charges are in red, and neutral charges are in purple. (**D**) Van der Waals and alkyl interactions with the residues of the catalytic site that are crucial for inactivating the enzyme.

According to earlier investigations, oleuropein (ligand) abundance over other phenolic components was observed in the HPLC chromatogram of olive leaf extract [25,26]. This compound was further selected for molecular docking studies to gain further insight into the molecular working mechanism by which lectin-ligand *(EFLec*–ligand) inhibits, as no published reports have described the protein-ligand structure. The result is shown in Figure 9.

*Enterococcus faecium* lectin (NCBI ID WP 033741131.1) has 11α helices and 7β strands. Auto lectin dock tools were used for molecular docking. Figure 9A shows *EFLec*–ligand binding modes as cartoons. Thr 244 (OH distance = 2.75), Gln305 (OH distance = 2.83), Gln305 (OH distance = 3.03), Gly245 (OH distance = 3.27), Gly258 (OH distance = 3.94), and Thr257 (OH distance = 2.82) formed hydrogen bonds (Table 1 and Figure 8B).

In the docked confirmation, the complete fitness and logarithm of lectin’s free binding energy (ΔG) were −1220.23 and −9.5 kcal/mol, respectively. Ligand and *EFLec* docking scores were also observed. Figure 9B shows the results. The predicted *EFLec* and ligand models for in silico docking were credible. *EFLec* active site amino acids were both high and less conserved. The conserved amino acids had ligand-binding motifs. Dimer binding motifs and surface residues were identified. All residues were allowed. Electrostatic forces were considered in the in silico docking experiments. Phe242, Thr244, Lys246, Glu255, Arg297, Asp301, and Gln305 formed hydrogen bonds.

In addition to carbohydrates, the ligand can interact with hydrophobic molecules in dimeric or tetrameric lectin structures, such as Ala232, Try254, Leu302, and Ile299. Figure 8B shows stable ligand binding. These hydrophobic interactions may explain why lectin Enterococcus faecium increases and decreases ligand activity [43,53]. A lectin-resveratrol phenolic compound interaction occurred in ConM’s hydrophobic cavity [54]. These results suggest that phenolic compounds extracted from plants, such as oleuropein, may be an alternative therapeutic tool to modify bacterial resistance. Half of the oleuropein locus was in the positive charge area during the in silico docking studies. Half was naturally electrostatic (Figure 9C). The results show that ligand interacted with *EFLec’s* active site through hydrogen bonding and van der Waals alkyl interactions (Figure 9D). These interactions inhibited *EFLec* activity. The ligand binded to the enzyme’s active pocket and interacted with the guanosine-5′-diphosphate active sites. The ligand competed with the substrate and created protein-product complexes as a competitive inhibitor.

### 2.5. Interaction Analysis of Ligand with Target EFLec

The ligand showed six interactions with the allosteric site residues of the target lectin protein. Six hydrogen bond interactions were observed, as shown in Table 2. The binding affinity energy (S-Score) of the allosteric site of the target protein was −9.5 kcal/mol.

**Figure 10:**
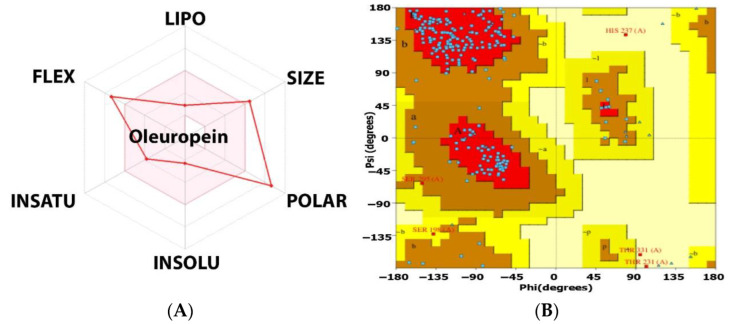
A three-dimensional interaction scheme of oleuropein with the catalytic site of *EFLec.* (**A**) Bioavailability radar related to physicochemical properties of molecules (criteria: MW 540.51 g/mol, fraction Csp3: 0.52, TPSA < 201.67 Å^2^, molar refractivity: 127.28, instauration, flexibility: 0.54 < rotatable bonds < 7), and (**B**) Ramachandran plot for the predicted model of the docking of oleuropein.

Figure 10A shows the bioavailability radar for a ligand with high activity potential; it meets all oral-use criteria (Table S1). The potent hits were physicochemical, pharmacokinetic, and drug-like (Bioavailability Source: 0.11, Ghose: NO, TPSA: 201.67 2, water solubility: 2.72 mg/mL soluble, BBB permeant: NO, P-gp substrate: NO, CYP isoform interacts: NO, oleuropein is water-soluble). Oleuropein does not cross the blood-brain barrier, does not interact with CYP450 enzymes, and is a P-gp substrate.

Ramachandran plots were utilised to verify the results. Overall, 100% of the residues were in the allowed area, while none were in the disallowed area. G-factors indicate a stereochemical property’s oddness. Below −1.0, the abnormality is high, and below −0.5, it is odd. G-factors for the main chain and dihedral angles were 0.5. According to the Ramachandran plot and G-factors, the 3D model’s backbone dihedral angles, phi, and psi were within permissible limits of the Programmer PROCHECK, RMSD, and ProSA (Figure 10B).

### 2.6. Molecular Dynamic Simulations

**Figure 11:**
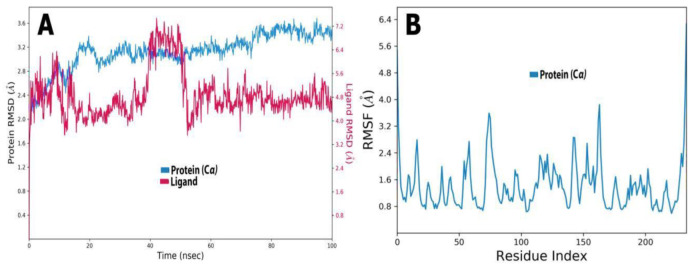
(**A**) RMSD plot obtained for oleuropein of the protein-ligand *EFLec* complexes. Protein Cα and compound RMSD are shown in blue and red colour, respectively. (**B**) RMSF plot of the protein’s backbone atoms during the 100 ns MD simulation. Protein Cα RMSD is shown in blue.

The inhibition of *EFLec* by ligands was assessed using an MD simulation and multiple dynamic trajectories (RMSD and RMSF; Figure 11A,B). Figure 11A shows a protein’s RMSD. Throughout the simulation, RMSD revealed the protein’s structure. RMSD analysis showed that oscillations near the MD simulation’s end were average thermal structures. Small and globular proteins, where MD simulation convergence is key, can tolerate 2.00–3.20 changes. RMSD should be a constant.

A study of the RMSF profile of the *EFLec* protein bound with oleuropein revealed fluctuations in the urease sequence. The RMSF profile for *EFLec* protein showed 1–2 nm fluctuations. Our study found no change at the *EFLec*–oleuropein active site. Non-catalytic site mobility was much higher, ranging from 4 to 50 nm, but this was not significant for the current study, which focused on the active site of *EFLec* (Figure 11B).

### 2.7. Ligand Torsion Profile and Properties

**Figure 12:**
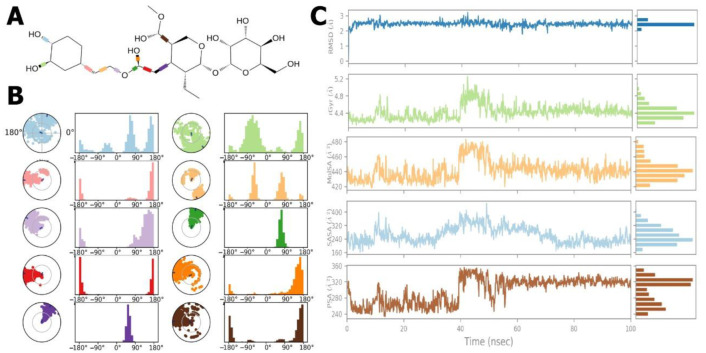
Ligand torsion profile: (**A**) torsion and flexibility, (**B**) ligand torsion angles, and (**C**) ligand (oleuropein) property trajectory of the ligand-*EFLec* complex. The ligand properties fluctuated during the beginning or intermediate simulation periods, but gradually returned to equilibrium during 100 ns of simulation, indicating that ligand was stable at the active site of the protein.

Figure 12A,B show the ligand torsion histogram throughout the simulation trajectory (0.00 ns to 100.05 ns). In Figure 12A, the ligand 2D-structure with colour-coded RB, as well as radial and bar plots for each RB torsion, are shown. The dial (or radial) graphs show the simulation’s torsion. The allosteric ligand was flexible, and the -OH side groups rotated along the simulated pathway. These data may reveal the ligand’s conformational strain to bind to protein (Figure 12B).

Figure 12C shows the examined ligand properties: RMSD, radius gyration, solvent-accessible surface area, molecular surface area, and polar surface area (PSA). The RMSD balanced to 100 ns. The ligand’s RSMD in reference conformation (time t = 0.00) varied from 1.00 Å^2^ to 2.00 Å^2^, and its balance was about 2.50 Å^2^.

Ligand length was measured in rGyr, or main inertia moment. According to the results, the rGyr showed ligand fluctuation until 40.00 ns of simulation, ranging from 4.20 Å^2^ to 5.20 Å^2^, and attaining equilibrium at 4.60 Å^2^.

The MolSA ligand was altered throughout the simulation. The ligand’s MolSA varied from 420 Å^2^ to 570 Å^2^ and achieved equilibrium at 450 Å^2^.

The SASA of the *EFLec*-ligand complex fluctuated until 40.00 ns during the MD simulation, then stabilized 170 Å^2^ to 380 Å^2^ and attained equilibrium at 280 Å^2^.

The PSA values of the ligand ranged from 240 Å^2^ to 360 Å^2^ until 40.00 ns and attained equilibrium at 320 Å^2^. Finally, all ligand attributes changed over the start or simulation period but found equilibrium at the conclusion. These results indicate that the ligand possessed a stable interaction to the active site of the protein.

**Figure 13:**
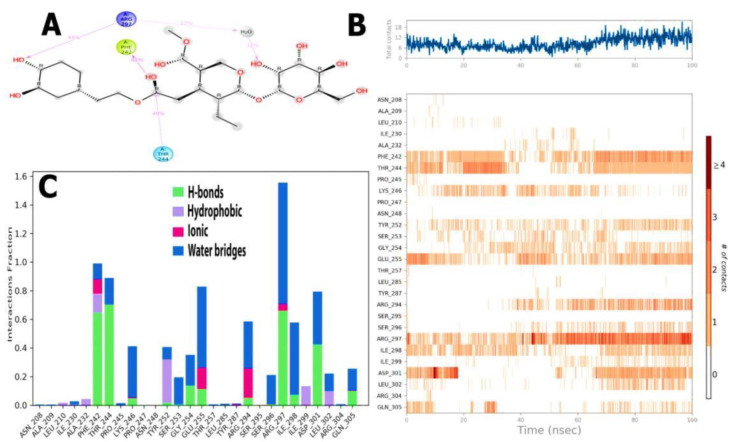
(**A**) Backbone RMSD values of the Cα atoms of the protein, (**B**) RMSF of the backbone atoms, (**C**) snapshots of the docking pose of oleuropein and the total H-bond intensity at various time intervals in the 20 ns complex MD simulations with *EFLec*.

H-bonds impact drug selectivity, metabolism, and adsorption in drug design. MD simulations boosted *EFLec*-ligand H-bond interactions while reducing the average total energy (Figure 13A,B). In silico analysis indicated that oleuropein’s docking conformation might build an H-bond with *EFLec’s* binding pocket residues. Ionic interactions occurred when two oppositely charged atoms came within 3.7 of one other, and no H-bond occurred. Phe242, Lys246, Glu255, Arg294, and Arg297 demonstrated little ligand-ionic interactions. Lau210, Ala232, Phe242, Ile299, Tyr252, and Leu302 reacted with aromatic or aliphatic ligand groups (Figure 13A). The water bridges represent H-bonded protein-ligand interactions mediated by water. Most major interacting amino acids (Ile230, Phe242, Thr244, Pro245, Lys246, Tyr253, Ser253, Gly254, Glu255, Arg294, Ser296, Ile298, Asp301, Leu302, Glu305, and Arg297) interacted through H-bonding and extremely efficient protein-ligand complex temporal interactions (100 ns simulation) (Figure 13B).

The ligand-receptor interaction histogram describes the protein-ligand interactions (Figure 13C). Figure 13C shows the number of protein-ligand interactions along the pathway. Above the trajectory curve series, interactions increased from 0.00 to 5.00. The role of amino acids in 100 ns MD simulations was studied. The orange scale below the graphic highlights residues that made several ligand contacts in a particular trajectory frame. The *EFLec* receptor-ligand complex showed deep orange bands (Phe242, Thr244, Lys246, Tyr252, Gly254, Glu255, Arg297, Ile298, and ArgAsp301 orange rows), suggesting that the amino acids exhibited excellent interactions with the ligand in almost all orientations (geometry). These results agree with our histogram findings.

## 3. Materials and Methods

### 3.1. Collection Samples

Fresh *Olea europaea* leaves were harvested with some branches from the Horticulture Department Medicinal Plants Garden of the Faculty of Agriculture, AL-Azhar University, Cairo, Egypt. The leaves were removed from the stems and cleaned by distilled water to remove residual dirt debris several times, then the leaves were left in the open air for a quarter of an hour in the shade until they dried.

### 3.2. Preparation of Olive Leaf Extract

Distilled water was used to extract phytochemicals from fresh leaves according to [55], with some minor modifications. In brief, the leaves were crushed and coarsely ground into a powder. The *OVLe* extraction was performed by soaking 1 g of the powder in 100 mL of deionized water. The solution was thoroughly mixed for 72 h and heated at 40 °C with continuous stirring using a magnetic stirrer. Then, the solution was left to cool down to room temperature. The colour of the extract was light greenish yellow. Finally, the extraction was filtered using (Whatman No.1) filter paper and kept at −20 °C in a volumetric glass vial to be employed in the green synthesis of copper nanoparticles.

### 3.3. Biosynthesis of CuO @OVLe NCs

*CuO @OVLe NCs* were synthesized using copper chloride as described by [56]. In brief, 40 mL of copper chloride (2 mm) was taken in an Erlenmeyer flask then mixed for almost 2 h. The OVLe mixture (1 mL) was subjected to the copper chloride solution under vigorous shaking (200 rpm) for 4 h at ambient temperature (25–27 °C) to permit the development of *CuO @OVLe NCs*. The solution soon became turbid and brown. To eliminate any contaminants or absorbed ions, the solution was centrifuged for 10 min at 12,000 rpm and rinsed with dH_2_O. Finally, the resultant was dried in the oven for 48 h at 45–50 °C with fan air.

### 3.4. Characterizations of CuO @OVLe NCs

Using the UV-Visible spectra, the optical characteristics of nanocrystal powder derived from the quantity of green production of copper oxide nanoparticles (*CuO @OVLe NCs*) were determined. The diluted samples were evaluated on a spectrophotometer (Varian Cary-100 Konc, Varian, Australia), at 230 V/50 Hz, at wavelengths of 200–800 nm. X-ray diffraction (XRD) patterns of the prepared *CuO @OVLe NCs* were recorded using the Rigaku D/Max-lllC X-ray diffractometer (Rigaku Int. Corp. Tokyo, Japan). It formed with a CuKa radiation set (20 mA and 40 kV) diffractions at a scanning rate of 20/min between 2 and 500 at ambient temperature. The FT-IR analysis was recorded for the prepared samples to investigate the functional groups using the JASCO FT-IR 4100 spectrometer (Hachioji, Tokyo, Japan) as potassium bromide (KBr) discs. A high-pressure disc was loaded and assessed with a resolution of 4.0 cm^−1^ at a wavelength of 400–4000 cm^−1^ [57].

### 3.5. Surface Morphology

Scanning electron microscopy (SEM) was used to image the optimised samples (JSM 6390^®^, JEOL DATUM Ltd., Tokyo, Japan). A drop of CuO @OVLe NCs dried for 5 min on the aluminium mesh below a mercury lamp coater to a thickness of 400 Å. For transmission electron microscopy (TEM), measurements were assessed by TEM (TOPCON002B; Tokyo, Japan). Equipment was used to examine the silver nanoparticle. By simply placing a small amount of the sample on the mesh and blotting away any surplus solution with blotting paper, thin films of silver nanoparticles were formed on a carbon-coated copper grid [58].

### 3.6. Culture of the Microorganism

Muller-Hinton agar medium was used to cultivate *Enterococcus faecium* for 24 h at 37 °C. Using UV-Vis spectroscopy (Varian Cary-100 Konc, Varian, Australia), the colonisation was collected and assembled in HBSS (pH 6.0) to 0.06 at 600 nm (OD_600_), which amounted to ~10^6^ (CFU) mL^−1^. It was then utilised in the examinations.

### 3.7. Antimicrobial Activity

The well diffusion method was used by producing three replications according to the method [59]. The bacterial suspension was prepared and dispersed throughout the medium on the medium of Muller-Hinton agar by means of a swab and afterward left for 5 min to dry. Then, 5 holes were made. Considering one of the pits as a conventional control, 50 µL of OVLe leaf extract (5, 10, 20, and 30 mg/mL) and *CuO @OVLe NCs* were attached in each pit at consecutive concentrations (10, 20, and 30 mg/mL), then put in the incubator for 24 h at 37 °C. DIH_2_O was employed as negative control, and tigecycline as a positive control. The inhibitory efficacy *CuO @OVLe NCs* against the bacteria was measured by the diameter of the inhibition region in millimetres using a standard ruler [60,61]. The outcomes are presented as mean ± standard deviation (SD).

### 3.8. Cell Lines

The normal liver cell line WRL 68 Cell as well as the cancer cell lines MCF-7,PC3 and HpeG2 were obtained from the University of Malaya College of Medicine Department of Pharmacy Center for Natural Product Research and Drug in Malaysia, Discovery Department of Pharmacology, Faculty Of Medicine, University of Malaya Kuala Lumpur Malaysia. Cancer cells were sustained and developed, and tests were conducted on them at the Biotechnology Research Center at Al-Nahrain University. All investigations were carried out using sterile deionized water (Milli-Q water).

### 3.9. Cytotoxicity Activity

The cytotoxic evaluation of the cancer cell lines MCF-7, PC3, and HpeG2 and normal liver cell line WRL-68 were evaluated with MTT. Following incubation, the medium from the wells was drained, and MTT solution (6 mg/mL) was added. The cells were placed in 60 µL of dimethylsulfoxide (DMSO), which was dissolved by rinsing the medium. The absorbance spectra of the specimens were identified using a microplate reader with a wavelength of 595 nm [62].

(1) % Cell viability =(A test − A blank)/(A control − A blank)

where A means optical density, test means the cells subjected to the *CuO @OVLe NCs* sample, control means the control sample, and blank means the wells without normal and cancer cells [63,64,65].

### 3.10. Molecular Docking

Structures in three dimensions of the citrus bioflavonoid oleuropein were downloaded from PubChem (ID 10621) [66]. They were docked onto the structure of the *Enterococcus faecium* lectin (NCBI ID WP 033741131.1) [67] using AutoDock Vina [68,69]. The docking pocket was determined using the default setting of AutoDock Vena. The utilised docking Grid Size was (Center X: 35.4 Y: 117.4 Z: 10.9), and dimensions (Angstrom) X: 41.8 Y: 42.9 Z: 45.3. The ligands were thought to be flexible, hence a ligand-specific torsion tree was created. Aside from that, the structure of protein was assumed rigid, and a grid covering the entire protein structure was established to represent the search area for possible inter-action sites. As a result, pre-calculated maps for the ligands were constructed, including separate maps for each atom in the ligand and incorporating desolvation together with electrostatic potentials. Furthermore, the Auto Grid technique was used to calculate the interaction energy of a ligand conformation by adding the contributions of atoms from a given element at any position in the grid near the stiff receptor. The docking results that passed a criterion of ΔG < −8.28 kcal/mol were sorted based on the predicted interaction energy after scanning the complete protein structure search space [70,71,72,73,74,75]. As a result, the highest-ranking ligand posture was picked as a projected interaction model. ADME profiling of oleuropein was performed according to [76].

### 3.11. Interaction of Oleuropein into the Active Pocket of ECLec

A technique for two-dimensional interaction of oleuropein together with the catalytic site of *ECLec* was designed by Discovery Studio 2019 software [77], Pymol software was used to generate the conservation score homology model and the electrostatic surface [78,79], pdbsum generated the Ramachandran plot for the anticipated model [80,81].

### 3.12. Molecular Dynamics Simulations (MD Simulations)

MD simulations were performed on the protein-ligand compounds utilising the Schrodinger Maestro package [82]. MD simulations of the *ECLec*-oleuropein docked complexes were carried out to illustrate the effectiveness of hesperidin in inhibiting lectin. The Lec topology file was created in the Gromacs software. The molecular dynamic simulation with a 2 fs time step use the charmm36 forcefield with TIP3P water model [83]. The pressure and temperature were measured using the Parrinello-Rahman algorithm and Nose-Hoover temperature coupling at 310 K and 1 bar, respectively. The simulations were run for 100 ns in the NPT collection. Per 20 ps, the trajectories are preserved. The visualization of trajectories was performed using VMD. The MD runs were accomplished on an Intel core I7 processor with 32 GB RAM, running a Linux operating system (Ubuntu v14.041) on a Thunderobot laptop.

### 3.13. Statistical Analysis

The results of statistical investigation are displayed as mean ± SEM. In contrast, one-way ANOVA determined statistical examination of data collected experimentally (* *p* < 0.05, ** *p* < 0.01, and *** *p* < 0.001) were regarded as significant differences when associated with the control. Graph Pad Prism 5.0 Version for Windows was used to evaluate statistical results (San Diego, CA, USA) [84,85].

## 4. Conclusions

Copper Bio-nanocrystals Oxide was successfully prepared using *Olea europaea* leaf extract as antimicrobial and anticancer reducing agents. When compared to chemical approaches, the applied method has various advantages, including ease of preparation, cost-effectiveness, and safety. The green synthesis process may be used to produce various metal oxide nanoparticles. UV/Vis, FTIR, XRD, EDX, SEM, and TEM were used to characterise *OVLe@CuO NCs*. The maximum UV/Vis wavelength was detected at 345 nm. The existence of colloidal *OVLe@CuO NCs* was verified by FTIR, XRD, and EDX. The *OVLe@CuO NCs* were found to be spherical and uniform in size, with an average size of 75 nm, as indicated by TEM and SEM. The nanoparticles inhibited the growth of VREfm and exhibited a potent anti-breast cancer activity. Further, a molecular docking simulation was used to demonstrate the catalytic activity of a ligand (oleuropein) with a lectin receptor complex of the outer membrane to VREfm with water solubility. The ligand exhibited P-gp substrate qualities according to the physicochemical, pharmacokinetic, and drug-likeness features of powerful hits. The MD simulations of these protein-ligand complexes demonstrated conformational alterations during ligand interaction with active site residues, as indicated by a comprehensive investigation of the dynamic trajectories and H-bond profiles throughout 100 ns of simulation. Taken together, our findings open new avenues for exploring safe and effective anti-*E. faecium* from *Olea europaea* leaf extract and developing novel pharmacologically active compounds by the green biosynthesis of metal nanocrystals.

## Figures and Tables

**Table 1:** Zone of inhibition (mm) plate with diverse concentrations of OVLe and *CuO @OVLe NCs*.

VREfm Zone of Inhibition (mm)			
Treatment	Inhibition Zone	Treatment	Inhibition Zone
OLVe 10 mg/mL	5.7	CuO@ OLVe 10 mg/mL	11.07
OLVe 15 mg/mL	6	CuO@ OLVe 20 mg/mL	18.02
OLVe 20 mg/mL	6.3	CuO@ OLVe 30 mg/mL	22.06
OLVe 25 mg/mL	6.5	Negative Control (Tigecyline, 25 µg/mL)	17.01
OLVe 30 mg/mL	7.2	Positive Control	NI

NI: No inhibition.

**Table 2:** Interaction analysis of ligand with target *EFLec*.

Ligand	Receptor/Amino Acid	Interaction Distance	E (kcal/mol)
O7/17	OG1/Thr244	H-donor 2.75	−1.2
O4/19	NE2/Gln305	H-acceptor 2.83	−2.5
O3/21	NE2/Gln305	H-acceptor 3.03	−1.2
O10/27	CA/Gly245	H-acceptor 3.27	−1.1
O10/27	CA/Gly258	H-acceptor 3.49	−0.9
O11/42	OG1/Thr257	H-acceptor 2.82	−2.6

## Data Availability

Not applicable.

## Supplementary Materials

The following supporting information can be downloaded at: https://www.mdpi.com/article/10.3390/molecules27227957/s1, Table S1: ADME predicted calculations of Oleuropein.

## Sample Availability

Samples of the nanoparticles are available from the authors.
