# Supplementary material for: Novel Copper Oxide Bio-Nanocrystals to Target Outer Membrane Lectin of Vancomycin-Resistant Enterococcus faecium (VREfm): In Silico, Bioavailability, Antimicrobial, and Anticancer Potential
Source: Molecules. 2022 Nov 17;27(22):7957. doi: 10.3390/molecules27227957 (PMC9696412; doi:10.3390/molecules27227957)
Supplement: Supplementary file 1 [file molecules-27-07957-s001.zip › molecules-1992167-supplementary.pdf]

Supplementary Information

Novel Copper Oxide Bio-nanocrystals to Target Outer Membrane Lectin of Vancomycin-Resistant *Enterococcus faecium* (VREfm): *In silico*, Bioavailability, Antimicrobial, and Anticancer Potential

Mahmoud Kandeel [1,2,*], Mohamed Sharaf [3,4,*], Arshad Mahdi Hamad [5], Ahmad O. Babalghith [6], Mohnad Abdalla [7,*], Muhammad Arif [4], Reem Binsuwaidan [8], Nashwah G. M. Attallah [9], Hossam Aladl Aladl Aladl [10], Samy Selim [11] and Mariusz Jaremko [12]

Table S1: ADME predicted calculations of Oleuropein

Physicochemical Properties	
Formula	C25H32O13
Molecular weight	540.51 g/mol
Num. heavy atoms	38
Num. arom. heavy atoms	6
Fraction Csp3	0.52
Num. rotatable bonds	11
Num. H-bond acceptors	13
Num. H-bond donors	6
Molar Refractivity	127.28
TPSA	201.67 Å²
Lipophilicity	
Log $P_{o/w}$ (iLOGP)	2.65
Log $P_{o/w}$ (XLOGP3)	-0.45
Log $P_{o/w}$ (WLOGP)	-0.63
Log $P_{o/w}$ (MLOGP)	-1.34
Log $P_{o/w}$ (SILICOS-IT)	-0.14
Consensus Log $P_{o/w}$	0.02
Water Solubility	
Log *S* (ESOL)	-2.30
Solubility	2.72e+00 mg/ml ; 5.03e-03 mol/l
Class	Soluble
Log *S* (Ali)	-3.32
Solubility	2.59e-01 mg/ml ; 4.79e-04 mol/l
Class	Soluble
Log *S* (SILICOS-IT)	-0.66
Solubility	1.19e+02 mg/ml ; 2.19e-01 mol/l
Class	Soluble
Pharmacokinetics	
GI absorption	Low
BBB permeant	No
P-gp substrate	No
CYP1A2 inhibitor	No
CYP2C19 inhibitor	No
CYP2C9 inhibitor	No

CYP2D6 inhibitor	No
CYP3A4 inhibitor	No
Log K_p (skin permeation)	-9.92 cm/s
Druglikeness	
Lipinski	No; 3 violations: MW>500, NorO>10, NHorOH>5
Ghose	No; 2 violations: MW>480, WLOGP<-0.4
Veber	No; 2 violations: Rotors>10, TPSA>140
Egan	No; 1 violation: TPSA>131.6
Muegge	No; 3 violations: TPSA>150, H-acc>10, H-don>5
Bioavailability Score	0.11
Medicinal Chemistry	
PAINS	1 alert: catechol_A
Brenk	3 alerts: catechol, isolated_alkene, more_than_2_esters
Leadlikeness	No; 2 violations: MW>350, Rotors>7
Synthetic accessibility	6.22
